# Canopy Vegetation Indices from *In situ* Hyperspectral Data to Assess Plant Water Status of Winter Wheat under Powdery Mildew Stress

**DOI:** 10.3389/fpls.2017.01219

**Published:** 2017-07-13

**Authors:** Wei Feng, Shuangli Qi, Yarong Heng, Yi Zhou, Yapeng Wu, Wandai Liu, Li He, Xiao Li

**Affiliations:** ^1^State Key Laboratory of Wheat and Maize Crop Science, National Engineering Research Centre for Wheat, Henan Agricultural University Zhengzhou, China; ^2^Collaborative Innovation Center of Henan Grain Crops, Henan Agricultural University Zhengzhou, China

**Keywords:** winter wheat, powdery mildew, hyperspectral remote sensing, photochemical reflectance index, plant water content

## Abstract

Plant disease and pests influence the physiological state and restricts the healthy growth of crops. Physiological measurements are considered the most accurate way of assessing plant health status. In this paper, we researched the use of an *in situ* hyperspectral remote sensor to detect plant water status in winter wheat infected with powdery mildew. Using a diseased nursery field and artificially inoculated open field experiments, we detected the canopy spectra of wheat at different developmental stages and under different degrees of disease severity. At the same time, destructive sampling was carried out for physical tests to investigate the change of physiological parameters under the condition of disease. Selected vegetation indices (VIs) were mostly comprised of green bands, and correlation coefficients between these common VIs and plant water content (PWC) were generally 0.784–0.902 (*p* < 0.001), indicating the green waveband may have great potential in the evaluation of water content of winter wheat under powdery mildew stress. The Photochemical Reflectance Index (PRI) was sensitive to physiological response influenced by powdery mildew, and the relationships of PRI with chlorophyll content, the maximum quantum efficiency of PSII photochemistry (Fv/Fm), and the potential activity of PSII photochemistry (Fv/Fo) were good with *R*^2^ = 0.639, 0.833, 0.808, respectively. Linear regressions showed PRI demonstrated a steady relationship with PWC across different growth conditions, with *R*^2^ = 0.817 and RMSE = 2.17. The acquired PRI model of wheat under the powdery mildew stress has a good compatibility to different experimental fields from booting stage to filling stage compared with the traditional water signal vegetation indices, WBI, FWBI_1_, and FWBI_2_. The verification results with independent data showed that PRI still performed better with *R*^2^ = 0.819 between measured and predicted, and corresponding RE = 8.26%. Thus, PRI is recommended as a potentially reliable indicator of PWC in winter wheat with powdery mildew stress. The results will help to understand the physical state of the plant, and provide technical support for disease control using remote sensing during wheat production.

## Introduction

Climate change is affecting crop disease prevalence, significantly influencing crop yield and quality worldwide (Christou and Twyman, 2004; Strange and Scott, 2005). Spectral remote sensing technology is used as a rapid, non-destructive, efficient method for detecting crop disease and evaluating the spatial variability across a crop (Liu et al., 2009). Effective evaluation of crop disease, accurate diagnosis and effective management is of great importance.

Disease stress generally results in changes in plant biomass and leaf structure, and a reduction in chlorophyll and water content. Blade shape and internal structural changes caused by disease ultimately change the reflective spectrum curve of plants (Jackson, 2003). Several techniques have been developed for detecting crop disease, such as fluorescence spectroscopy (Sindhuja et al., 2010), and remote sensing (Zhang et al., 2005; Sankaran et al., 2010; Dammer et al., 2011; Usha and Singh, 2013). Band combinations from hyperspectral imaging have generally been applied to detect plant disease (Larsolle et al., 2007). Several studies have shown that the most sensitive bands for crop disease identification are commonly located in the visible and the near infrared region (Wang et al., 2002; Bravo et al., 2003; Cheng et al., 2010), however, the optimal sensitive wavebands depend on how the disease interacts with a specific crop species (Mahlein et al., 2013). Green bands can effectively monitor yellow rust at wheat canopy level (Huang et al., 2007; Zhang et al., 2012b). Previous studies have revealed the role of red-edge, green, and blue bands to detect laurel wilt on avocado (Castro et al., 2015). Some studies established different vegetation indices (PMI, PRI, DGVI) to establish plant disease severity (Huang et al., 2007; Mahlein et al., 2013; Feng et al., 2016). Other studies applied multivariate analysis tools to detect spectral changes and disease development, such as neural networks (Castro et al., 2012), partial least squares regression (Zhang et al., 2012a,b, 2014), factor analysis and back propagation neural network (Shen et al., 2015b). Above studies showed that different spectral analysis methods can monitoring plant disease on a real-time basis, which could provide a theoretical platform for precise control of pests and diseases.

During pathogenic attack, enzyme activity is released to hinder the pathogen, cell energy metabolism and function is affected, and the internal physiological state of the plant and leaf changes. The spectral reflectance after pathogenic attack increases at visible and short-wave infrared, and reduces at the near infrared band; the typical spectral characteristics differ greatly with a healthy crop (Cheng et al., 2010; Zhang et al., 2012a). Previous research on disease severity levels focus less on the plant physiological state. Liu et al. (2014) used a simple ratio of reflectance at 890–780 nm in the near-infrared shoulder region for evaluating blade structure deterioration under herbicide injury or stripe rust in winter wheat. Feng et al. (2013) research showed that the Normalized Difference Angle Index (NDAI) was reliable for estimating changes in chlorophyll density following infection of the wheat canopy. Xu et al. (2011) and Chen et al. (2010) continued to estimate the changes in chlorophyll content under disease and insect infection in plants. These findings suggest that remote identification of plant physiological parameters after infection is feasible, and is a rapid and convenient evaluation of the physiological response to crop disease.

There has been great progress on monitoring plant water status using remote sensing mostly based on the water sensitive absorption band signal from hyperspectral remote sensing data. The fixed-position Water Band Index (WBI) positively correlates with water content in beans (Xu et al., 2007), and the Floating-position Water Band Index (FWBI) has been shown to correlate with area-weighted water content of leaf in corn (Strachan et al., 2002). Meanwhile, some researchers estimated the water status of plants, not based on the moisture absorption band, but xanthophyll cycle pigments related to heat dissipation. The Photochemical Reflectance Index (PRI) has been proposed to evaluate vegetation water stress at the leaf level (Penuelas et al., 1997b; Suárez et al., 2008, 2010; Neues et al., 2009; Zarco-Tejada et al., 2012). Nevertheless, Thenot et al. (2002) revealed PRI sensitivity to water stress conditions, despite the structure effect caused by water pressure affecting the reflected signal. Dobrowski et al. (2005) and Evain et al. (2004) shows that PRI can accurately track induced water stress conditions. The studies were carried out under abiotic stress to estimate vegetation water content using spectroscopy technology. However, under biological stress, the plant physiological state will significantly change, for instance, bacterial plaque coverage, increased leaf yellowing, canopy structure destruction, altering the signal. Therefore, the WBI may not be suitable for testing the dynamics of moisture under abiotic stress which require xanthophyll cycle pigments to detect moisture.

This study aims to evaluate the potential for using PRI as an indirect indicator of plant water content (PWC) at canopy scale in winter wheat under powdery mildew stress. The objectives of this study were to (1) analyze the traditional water vegetation indices (VIs) to determine canopy PWC of winter wheat in a diseased field and artificially inoculated open field; (2) assess the response of leaf chlorophyll content (Chl content) and PWC under powdery mildew stress; (3) quantify the relationship between PRI and disease index (DI), leaf Chl content, and fluorescence parameters and (4) evaluate the method using PRI to measure water conditions under powdery mildew stress based on the ground-based canopy hyperspectral technology. The results will provide a basis for disease grading and classification, and further provide technical support for early recognition and precise control during crop production.

## Materials and methods

### Experimental design

These experiments were conducted in the wheat growing seasons of 2014–2016, located in Zhengzhou, China. The powdery mildew susceptible varieties, Yanzhan 4110, was selected as the test material. The field site consisted of loam soil. The 0–30 cm soil basic indicators were total N 0.97–1.20 g kg^−1^, available phosphorus 28.44–34.52 mg kg^−1^, available K 114.68–120.12 mg kg^−1^, and organic matter 10.2–15.7 g kg^−1^ pre-planting. Nitrogen fertilizer application (135 kg hm^−2^ nitrogen) in the study plots was higher (12.5%) than the local field practice, plus 120 kg hm^−2^ P_2_O_5_, and 90 kg hm^−2^ K_2_O as basic fertilizer. A second dose of nitrogen fertilizer (135 kg hm^−2^ nitrogen) was combined with the irrigation at the jointing stage. The study plots were watered 3 times at the jointing stage, booting stage, and flowering stage, and the corresponding local fields were watered 2 times at jointing stage and flowering stage. Other aspects of field management followed standard local practices for wheat production.

Experiment 1 was carried out at a diseased nursery field located in the experimental station of National Engineering Research Center for Wheat, (34°47′ N, 113°39′ E). Wheat was sown on 22 October 2014, in the north-south direction with 20 cm row spacing, and the area of the plot was 8.2 m^2^ (2.1 × 4 m). Experiment 2 was carried out at an open field located in the experimental farm of Henan Agricultural University (34°51′ N, 113°35′ E). Wheat was sown on 15 October 2014, and each plot area was 20.3 m^2^ (7 × 2.9 m) during the 2014–2015 growing season. There were four disease treatments with three replications. The healthy treatment was not inoculated, the low level treatment was inoculated every 4 days, the medium level was inoculated by every 2 days, and the severe treatment were inoculated every day. Experiments 3 and 4 were repeated trials of Experiments 1 and 2 at the same location in the following year. Wheat was sown on 22 October 2015 and 12 October 2015, respectively, during the 2015–2016 growing season. To avoid powdery mildew cross-contamination between plots, powdery mildew resistant triticale was sown between experimental plots. Each plot was divided by a 0.6 m spacing which contained two rows of triticale. Triticale has a plant height of 2 m. The data from Experiments 1–3 were used to build the model and the independent Experiment 4 was used to test the model.

**Figure 1:**
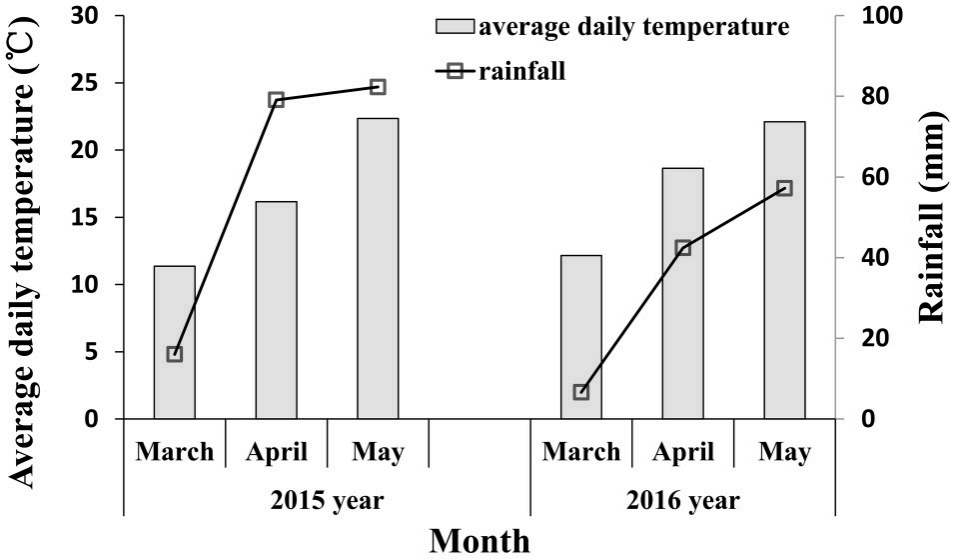
Meteorological data of March to May in 2015–2016 under our experimental area.

**Figure 2:**
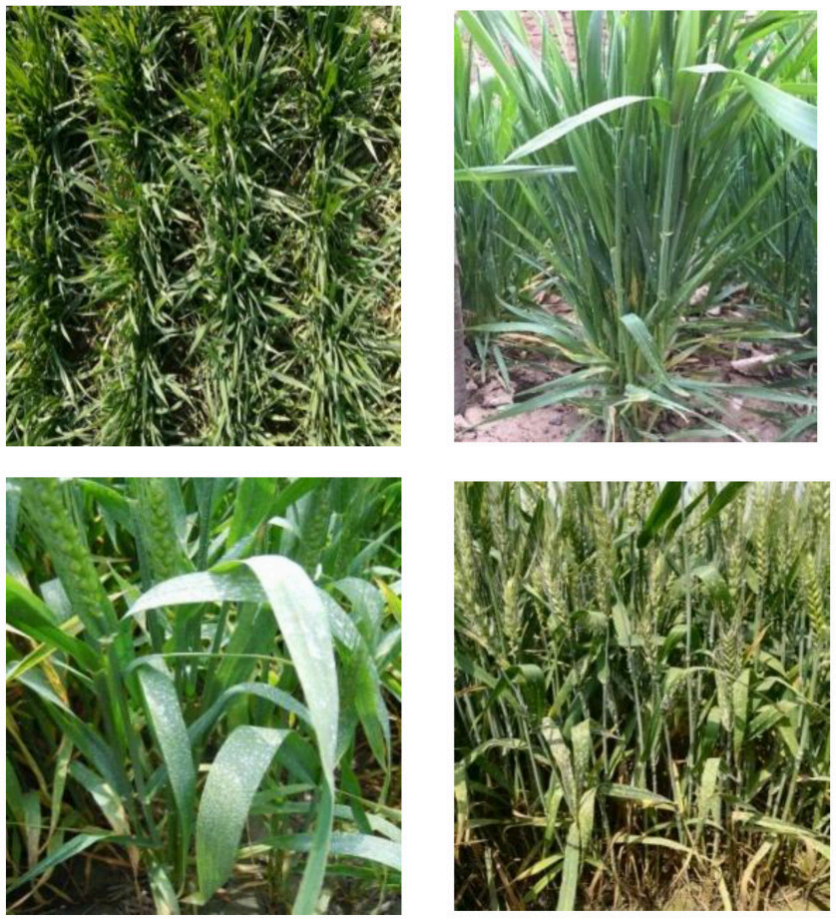
Pictures of the different developmental stages of the infected winter wheat; jointing **(left upper)**, booting **(right upper)**, anthesis **(left lower)**, and filling **(left lower)**.

Infected plants with soil were excavated and placed into prepared pots (18 cm in diameter, 15 cm in height) for inoculating the field experiments from the jointing stage. After 16:00, infected plants with powdery mildew fungi were brought to the inoculated field. The infected plants were shaken above the experimental plot to infect the wheat and then the infected plants were placed in the center of the experimental plots. The inoculated plot was covered with a transparent shed which remained open in the day and closed at night; the inside of shed was kept moist and the temperature at night was 10°C. After 10 days, symptoms began to appear, and once the wheat infection became obvious during the late jointing stage, the shed was removed. The meteorological data during powdery mildew infection are shown in Figure 1. Rainfall in 2015 was higher than in 2016, especially in April (corresponding to the booting and flowering stage of wheat) and May (corresponding to filling stage of wheat), which is conducive with the occurrence and spread of wheat powdery mildew. The key growth period of wheat is shown in Figure 2. A representative area in each plot was selected to investigate the severity of the disease, measure the reflectivity of the canopy and chlorophyll fluorescence parameters, and determine plant chlorophyll content at booting stage, anthesis, and filling stage.

### Data collection

#### Measurement of *in situ* canopy reflectance spectrum

Wheat canopy reflectance spectra were measured by FieldSpec HandHeld spectrometer (Analytical Spectral Devices Inc., Boulder, Colorado, USA) over 350–2,500 nm spectral region, at 0.5 m above the wheat canopy with a field of view of 25° (Cao et al., 2013), and a spectral sampling interval of 1.4 nm for the 350–1,050 nm region and 2 nm for the 1,000–2,500 nm region. Each spectrum measurement was carried out under sunny and windless conditions at 10:00–14:00 local time. For each sampling point, a view area of ~0.17 m^2^ of wheat canopy was selected to measure canopy reflectance spectra, five sequential readings were averaged to obtain one spectrum, and to analyze biochemical and biophysical canopy features. Instrument optimization was performed prior to sample acquisition, with the average of 50 dark current and white reference, respectively. A white BaSO_4_ calibration panel was taken before canopy measurements to calculate baseline reflectance. DC measurement can be updated at any time, but should be updated more frequently in the beginning of a given session, as the instrument warms up. Finally, reflectance spectra were obtained by determining the ratios of data acquired for a sample to data acquired for the white reflectance standard.

#### Measurement of chlorophyll fluorescence parameters

Chlorophyll fluorescence parameters were measured on the level of single leaves using a modulated fluorimeter (MiniPAM Photosynthesis Yield Analyser, Walz, Effeltrich, Germany). Fluorescence parameters were measured at 10:00–11:30 local time. The minimum fluorescence (Fo) was induced by a weak pulse of modulating light applied (<0.3 μmol photons m^−2^ s^−1^), which ensured that almost all the PSII reaction centers were in the open state. The maximum fluorescence (Fm) was determined by saturating pulse light of 8,000 μmol photons m^−2^ s^−1^ over a 0.8 s period (reaction centers fully closed). Both the values of Fo and Fm were measured on a leaf under full dark-adaptation for 30 min. The difference between Fm and Fo generated the variable fluorescence (Fv, Fv = Fm−Fo). The maximum quantum efficiency of PSII (Fv/Fm, Fv/Fm = (Fm−Fo)/ Fm) has been widely used as a sensitive indicator of plant photosynthetic performance (Shirke and Pathre, 2003) and detect stress-induced perturbations in the photosynthetic apparatus (Baker and Rosenqvist, 2004). The maximum primary yield of photochemistry of PSII (Fv/Fo, Fv/Fo = (Fm−Fo)/ Fo), showed a close relationship with Fv/Fm (Li et al., 2006; Sharma et al., 2014). The Fv/Fo has been interpreted as an indicator of the structural alterations on the donor side of the PSII (Christen et al., 2007), and it decreased with increasing of temperature (Janka et al., 2013), drought stress (Li et al., 2006), aluminum stress (Peixoto et al., 2002) and nitrogen deficiency (Lima et al., 1999).

#### Assessment of disease index

In each spectral test location, 20 plants of wheat were randomly selected to measure the incidence of wheat powdery mildew. To reduce human error, all the tests were conducted by the same person under the guidance and supervision of professionals who major in plant protection.

The severity of all fully expanded leaves was used to indicate the incidence condition of the sampling point, and the grid method (lesion area to total fully expanded leaf area percentage) to calculate the severity of powdery mildew. Powdery mildew severity was divided into nine grades, as follows: 0, 1, 10, 20, 30, 45, 60, 80, and 100%. The DI was computed using the following formula (Cai et al., 2007).

$$\text{DI}=\frac{\sum \text{xf}}{\text{n}\sum \text{f}}\times 100$$

Here, x is the value of each incidence level, n is the value of the highest level (*n* = 9) of powdery mildew incidence, and f is the value of total leaves in each level of disease severity.

#### Measurement of leaf chlorophyll content and plant water content

Physiological data measurements were required at the spectral sampling points. After each canopy spectral reflectance and leaf chlorophyll fluorescence measurement, 10 plants from each plot were selected randomly and destructively sampled for plant fresh weight (W_F_). The sample was then denatured at 105°C for 30 min and at 80°C until a constant weight (dry weight, W_D_). PWC was calculated using the method of weight ratio (Jones et al., 2004):

$$PWC(\%)=(W_{F}-W_{D})/W_{D}\times 100$$

For each treatment, three wheat stems showing typical symptoms were selected. From these three stems, nine leaf samples were taken from the second, third and fourth leaf from the top of wheat plant. The veins were removed and 0.2 g cut from each sample. The samples were ground with 10 ml acetone at 80%, before adding 40 ml acetone to a total of 50 ml in a brown volumetric flask. These flasks were stored in the dark at 4°C for 48–72 h, ensuring complete chlorophyll extraction. Absorption was measured at 470, 649, and 665 nm with a spectrophotometer. The average value of three replicates was used to calculate the concentrations (mg/g fresh leaf mass) of leaf chlorophyll *a* and chlorophyll *b* using the formula described by Lichtenthaler (1987).

### Data analysis

Several spectral indices previously reported in the literature were selected to check the correlation with PWC; optimized VIs are listed in Table 1. The best equations were established between physiological parameters, DI and spectral parameters by regression under powdery mildew stress of wheat. Equation evaluation was optimized through *R*^2^ and a self-developed computer program based on the software of MATLAB 7.0. Data from Experiments 1–3 were used to establish the relationship between the spectral indices and PWC. Data from Experiment 4 were used to validate the PWC monitoring models; the estimated values were compared with the measured values to assess reliability and accuracy of the equation output under actual conditions. Root mean square error (RMSE), relative error (RE), and precision (*R*^2^, the determination coefficients between the measured and estimated values) were applied to check the applicability between the estimated and measured data. RMSE and RE obeyed the following Equations (1) and (2), respectively:

(1) $$\text{RMSE}=\sqrt{\frac{1}{n}\times \sum_{i=1}^{n}{\left(P_{i}-O_{i}\right)}^{2}}$$

(2) $$\text{RE(}\%\text{ )}=\sqrt{\frac{1}{n}\times \sum_{i=1}^{n}(\frac{P_{i}-O_{i}}{O_{i}})\times 100\%}$$

Where, P_i_ and O_i_ are the estimated and measured values, respectively, and n is the sampling number. Estimation was superior if RE < 10%, good if RE = 10–20%, and acceptable if RE = 20–30% (Feng et al., 2014).

**Table 1:** Summary of selected spectral parameters reported in the literature.

**Spectral parameters**	**Formula or depiction**	**References**
Water band index(WBI)	WBI = R_950_/R_900_	Xu et al., 2007
Floating-position water band index (FWBI_1_)	FWBI_1_ = R_900_/min (R_930−980_)	Strachan et al., 2002
Floating-position water band index (FWBI_2_)	FWBI_2_ = R_920_/min (R_960−1000_)	Harris et al., 2006
Visible atmospherically resistant index (VARIgreen)	VARIgreen = (R_Green_-R_Red_)/(R_Green_+R_Red_−R_Blue_)	Gitelson et al., 2002
Simple Ratio Pigment Index (SRPI)	SRPI = R_430_/R_680_	Penuelas et al., 1994
Ratio vegetation index (RVI)	RVI = R_493_/R_678_	Tilley et al., 2003
Ratio index of the double-peak areas (RIDA)	$\text{RIDA}=\int_{k}^{755}\frac{dR\lambda }{d\lambda }d\lambda /\int_{680}^{k}\frac{dR\lambda }{d\lambda }d\lambda$	Feng et al., 2014
Red green ratio chlorophyll content (RGRcn)	RGRcn = (R_612_+R_660_)/(R_510_+R_560_)	Steddom et al., 2003
Lo_min_	corresponding wavelength of minimum band reflectance ranging from 640 to 680 nm	Chen et al., 2011
Anthocyanin index (AI)	AI = R_(600−699)_/R_(500−599)_	Gamon and Surfus, 1999
R_705_/(R_717_+R_491_)	R_705_/(R_717_+R_491_)	Tian et al., 2011
Photochemical reflectance index (PRI)	PRI = (R_570_−R_531_)/(R_570_+R_531_)	Gamon et al., 1992

*R is the reflectance at a given wavelength. For example, R_900_ is the spectral reflectance value at 900 nm*.

## Results

### Canopy reflectance spectra with different stages and disease indexes

**Figure 3:**
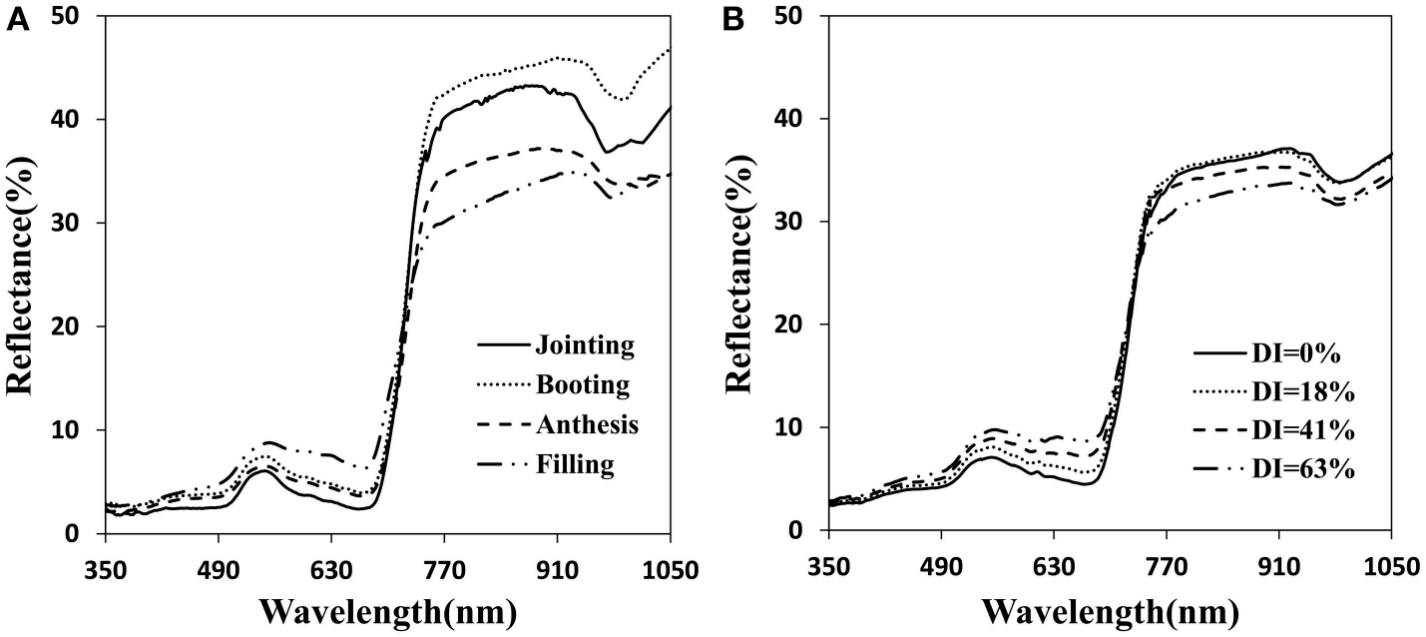
Canopy reflectance spectra for the different infected developmental stages **(A)** and for different disease index of powdery mildew at anthesis **(B)**.

The canopy reflectance was distinctly different at each growth period and disease severity (Figure 3). The initial symptoms of the disease occurred mainly in the lower portion of the plant. With disease progression, symptoms began to appear obvious in middle and upper layers. Generally, with increasing growth period, the reflectance tended to increase in the visible region and initially increased then decreased in the near-infrared region (Figure 3A). The severity of the powdery mildew affected the reflectance spectra of the canopy similar to the growth stages in the visible region, but decreased reflectance in the near-infrared wavebands as the disease worsened (Figure 3B). By comparison, visible wavebands are more sensitive to disease severity than near-infrared wavebands, and sensitive wavebands are mainly between 500 and 670 nm. Increased canopy coverage and strong chlorophyll absorption resulted in a decrease in canopy reflectance in the visible region. Reflectance in the near-infrared region may have been caused by *Sphaerotheca fuliginea* cover on the leaf surface, structural damage of the leaf surface, yellowing and decreased water content, etc.

### Relationship between traditional water band index and plant water content

**Figure 4:**
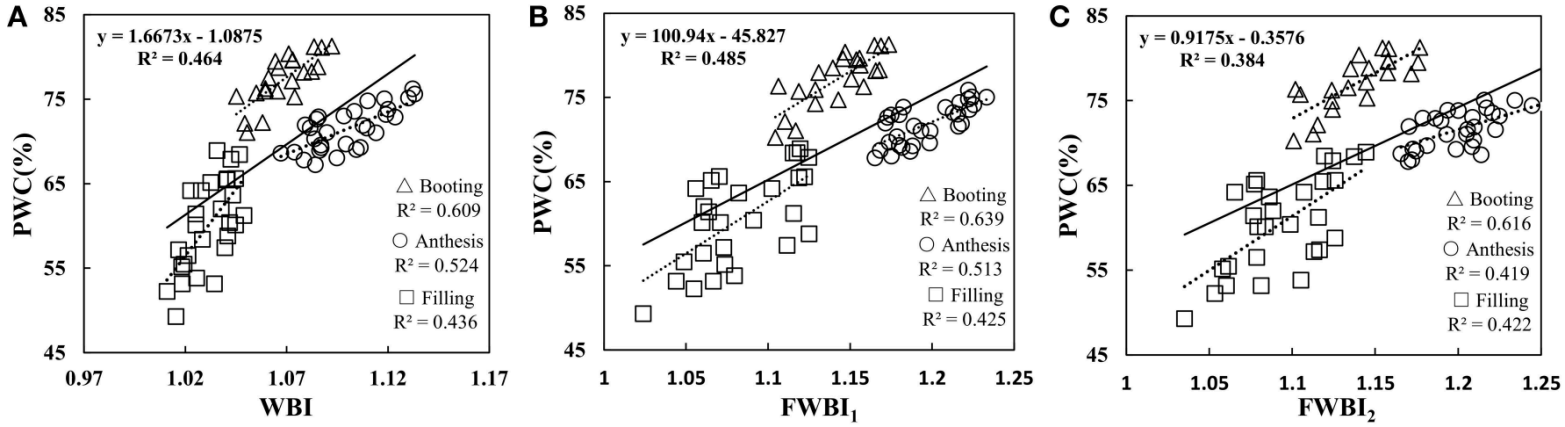
Relationship between **(A)** WBI, **(B)** FWBI_1_, and **(C)** FWBI_2_ with PWC. Data points from Experiments 1–3 (*n* = 82).

To understand whether the reported water band indices effectively monitored the water dynamic change under disease stress in winter wheat, we performed correlation between representative water indicator and PWC at booting, anthesis, and filling stage using data from Experiments 1–3 (Table 2). The linear relationships of WBI, FWBI_1_ and FWBI_2_ to PWC were relatively poor (*R*^2^ = 0.38–0.49) across each developmental stage used for reflectance data acquisition (Figure 4). Furthermore, individual regression analysis was conducted using specific growth period datasets. The accuracy of the regression model was significantly affected by developmental stage. The three typical water indices showed significantly different correlation with PWC at different growth stages (*R*^2^ = 0.41–0.64). These water band indices were linearly correlated with PWC at the booting stage (*R*^2^ = 0.60–0.64), but less so at flowering and filling stages (*R*^2^ = 0.41–0.53 at flowering stage; 0.42–0.44 at filling stage). Typical water band indices were unsuitable across different stages for detection of water status under powdery mildew stress, and this may be due to significant superimposed effects from physiological and biological changes under biotic stress, and which requires us to seek out efficient indicator of physiological parameters under disease stress.

**Table 2:** Correlation coefficients between canopy spectral parameters and plant water content (PWC) of winter wheat infected with powdery mildew.

**Spectral parameters**	**Experiment**			**Growth Stages**			**All**
	**Exp. 1**	**Exp. 2**	**Exp. 3**	**Booting**	**Anthesis**	**Filling**	
WBI	0.645^***^	0.764^***^	0.665^***^	0.781^***^	0.724^***^	0.660^***^	0.681^***^
FWBI_I_	0.685^***^	0.783^***^	0.635^***^	0.799^***^	0.716^***^	0.652^***^	0.696^***^
FWBI_2_	0.571^**^	0.702^***^	0.618^***^	0.748^***^	0.799^***^	0.650^***^	0.618^***^
VARI_*green*_	0.864^***^	0.752^***^	0.822^***^	0.492^*^	0.754^***^	0.771^***^	0.789^***^
SRPI	0.845^***^	0.75^***^	0.811^***^	0.582^**^	0.567^***^	0.811^***^	0.819^***^
RVI_(493, 678)_	0.877^***^	0.708^***^	0.734^***^	0.541^**^	0.678^***^	0.828^***^	0.803^***^
RIDA	0.893^***^	0.883^***^	0.653^***^	0.375	0.633^***^	0.735^***^	0.802^***^
RGR_cn_	0.861^***^	0.776^***^	0.835^***^	0.566^**^	0.741^***^	0.747^***^	0.816^***^
Lo_min_	0.913^***^	0.902^***^	0.636^***^	0.521^*^	0.714^***^	0.733^***^	0.792^***^
AI	0.884^***^	0.769^***^	0.809^***^	0.413	0.741^***^	0.727^***^	0.793^***^
R_705_/(R_717_+R_491_)	0.886^***^	0.813^***^	0.654^***^	0.633^**^	0.659^***^	0.775^***^	0.784^***^
PRI	0.917^***^	0.835^***^	0.853^***^	0.815^***^	0.869^***^	0.922^***^	0.902^***^

* *p < 0.05*,

** *p < 0.01*,

*** *p < 0.001*.

### Correlation between common vegetation indices and plant water content

Previous VIs were developed mostly under abiotic stress. Nine out of the 12 published VIs in Table 1 were not combination with water absorption wavebands, and then selected to examine estimation strength of PWC using MATLAB 7.0 software. These nine VIs were significantly correlated with PWC using whole datasets, single stage datasets or single experiment datasets (Table 2). This correlation analysis showed that PRI was superior to other common indices in terms of accuracy and ability to track PWC changes in winter wheat under disease stress. Population correlation coefficient of PRI and PWC was 0.902 (*p* < 0.001). The four VIs of SPRI, RGRcn, RIDA and RVI (493,678) were followed to give better correlation (*r* > 0.8).

**Figure 5:**
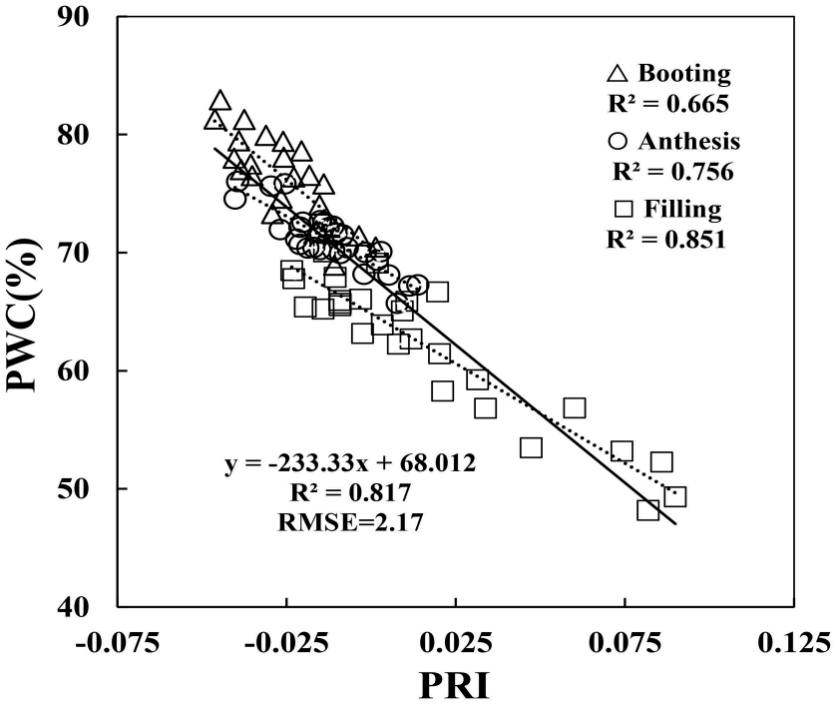
Relationship between PWC and PRI. Data points from Experiments 1–3 (*n* = 82).

Since the collected data was from two different types of test systems and three growth stages, correlation between VIs and PWC varied according to different growth conditions. By comparison, correlation in Experiment 1 was better than the other two experiments, and correlation at booting stage was poorer than the other two stages. For example, at booting stage, both RIDA and AI with PWC show no significant correlation at the 0.05 probability level (*r* < 0.413), VARIgreen and Lo with PWC were generated the weakest significance correlation at the 0.05 probability level (*r* < 0.521), and other VIs with PWC gave higher significance correlation at the 0.01 probability level (*r* < 0.815). As shown in Figure 5, the regression equations for PWC with PRI well illustrates the dynamic pattern of PWC changes in winter wheat which suffer from powdery mildew with *R*^2^ values of 0.817 and RMSE of 0.217, minimizing the possible heterogeneity from different growth stages.

### The effects of disease severity on plant water content and leaf chlorophyll content

**Figure 6:**
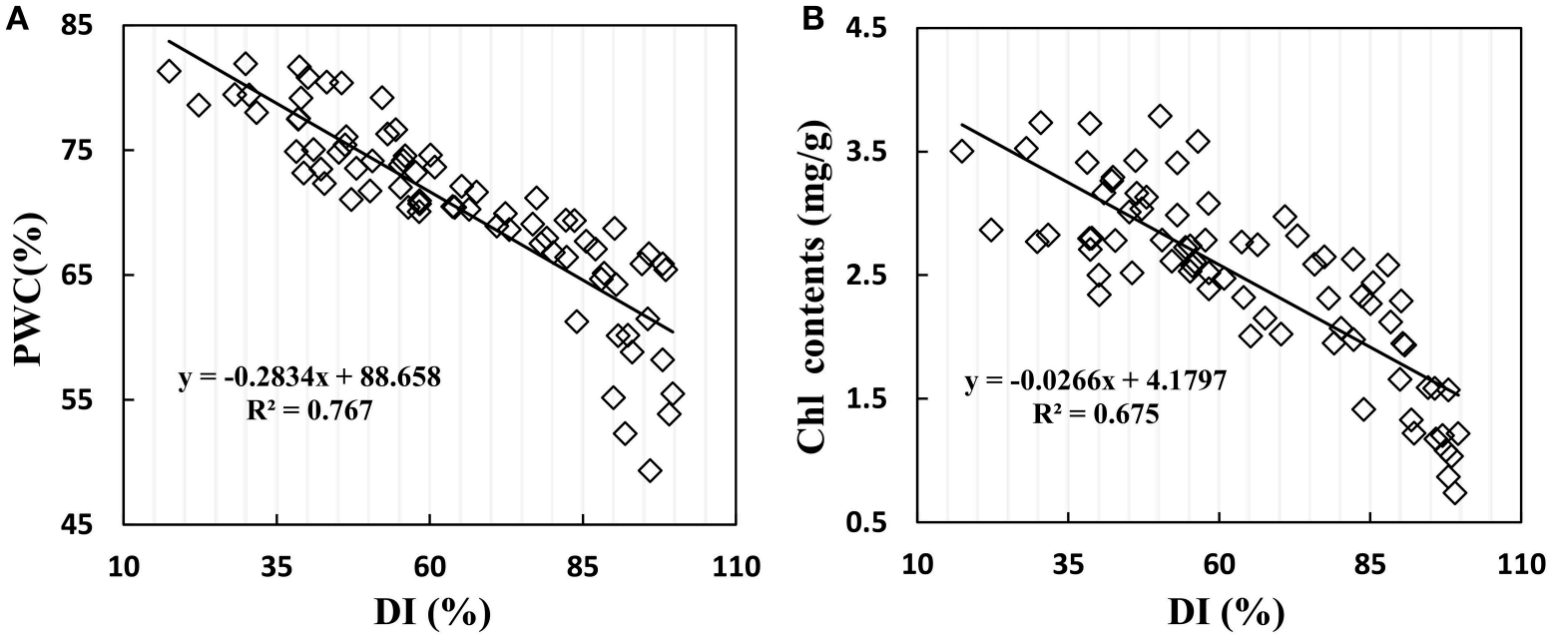
Relationship between **(A)** PWC, **(B)** leaf chlorophyll contents and disease index (DI) in winter wheat. Data points from Experiments 1–3 (*n* = 82).

Disease stress affected crop physiological processes in a complex way, and the linear regression showed that physiological parameters of PWC and Chl content are sensitive to disease severity in winter wheat. When suffering from powdery mildew damage in winter wheat, the early symptoms are not obvious; formation of suborbicular disease spot, and the physiological process only change slightly. With increasing disease, wheat growth parameters take on significant changes with reduction of biomass and deterioration of leaf tissue structure. Figure 6 shows the close relationship between growth parameters and disease severity, with *R*^2^ values of 0.767 for PWC and 0.675 for Chl content, indicating serious and stable effects of disease severity on PWC and leaf Chl content in winter wheat.

### Relationship between photochemical reflectance index and disease index, chlorophyll content and fluorescence parameters

**Figure 7:**
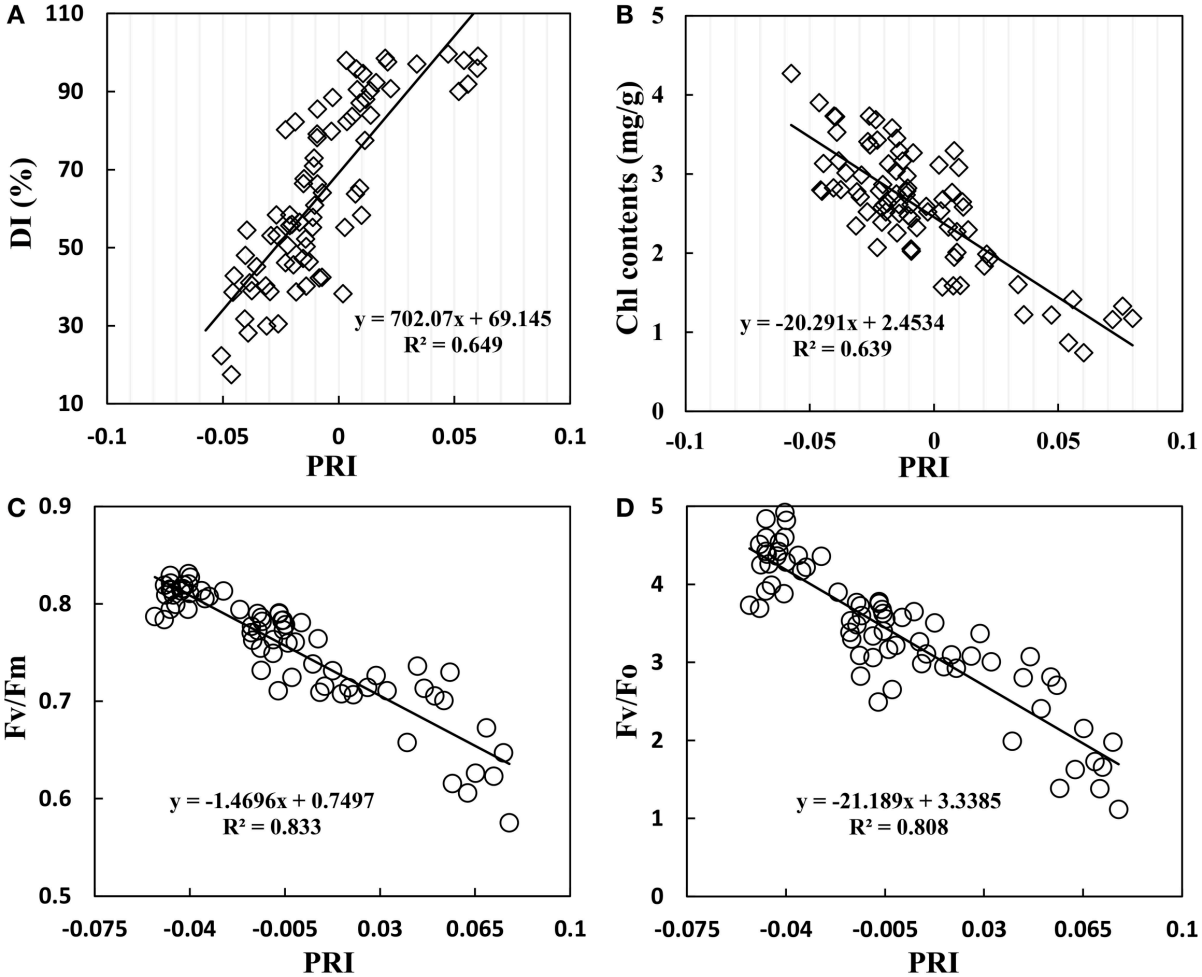
Relationship between DI **(A)**, leaf chlorophyll contents **(B)**, F_v_/F_m_ (maximum photochemical efficiency of PSII) **(C)**, F_v_/F_o_ (potential activity of PSII) **(D)**, and the photochemical reflectance index (PRI) in winter wheat. Data points from Experiments1–3.

The infection of powdery mildew not only affected wheat growth parameters, but also lead to change in canopy reflectance. As shown in Figure 7A, PRI gradually increased with increasing disease severity; there was a significant positive correlation with *R*^2^ of 0.649. However, PRI showed a significant negative correlation with Chl content of *R*^2^ = 0.639 (Figure 7B). Chlorophyll fluorescence is an important parameter to assess photosynthetic apparatus, particularly PSII activity in response to environmental stresses. A negative correlation was found between PRI and chlorophyll fluorescence parameters in wheat leaves, with an *R*^2^ of 0.833 for Fv/Fm (Figure 7C) and 0.808 for Fv/Fo (Figure 7D). This close linear relationship between chlorophyll fluorescence parameters and PRI showed a homogeneous pattern between efficiency of solar energy utilization and photochemical activity of PSII system, indicating that PRI agreed well with photosynthetic capacity of plants under powdery mildew stress.

### Testing photochemical reflectance index and plant water content relationship model

**Figure 8:**
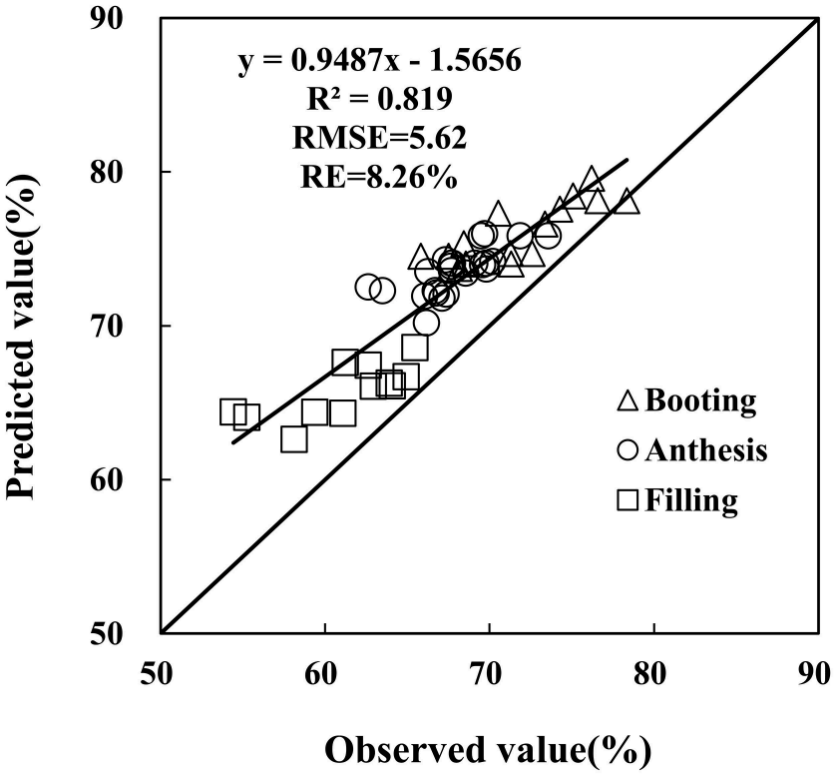
Comparison of predicted and observed wheat PWC based on the PRI in winter wheat. Data points from Experiment 4 (*n* = 52).

The monitoring model of PWC has been set up through hyperspectral indices from remote sensing data, yet further examination is required on the reliability and generalization of the PRI-PWC regression equation using the independent dataset obtained from Experiment 4 (*n* = 52). The 1:1 relationship between the observed and estimated values confirmed the accuracy of the derived model (Figure 8). The *R*^2^ values between measured and estimated PWC were 0.819, generating RMSE of 5.62 and RE of 8.26%, and the predicted values were in general higher than the measured values. The above results suggest that the PRI model performs better for detecting PWC under powdery mildew stress.

## Discussion

The sensitivities of the visible and near-infrared wavebands to the disease were different depending on disease type (Mahlein et al., 2013). Studies have shown that the change of spectral reflectance was more obvious in visible wavebands than in the near-infrared wavebands under disease stress (Cheng et al., 2011; Zhang et al., 2011). The first symptom of powdery mildew infection is formation of suborbicular disease spot; as the disease develops, the leaf surface becomes covered by a layer of the mold (Allen, 1942). Following powdery mildew infection, wheat growth parameters change significantly with a decrease in dry matter accumulation, Chl content and moisture content, as well as deterioration of leaf tissue structure. However, crop canopy reflectance showed a steady increase in the red wavebands (Cheng et al., 2010; Chen et al., 2011). The spectral reflectance demonstrated a strong correlation with disease severity, and sensitive wavebands were mostly located in the green region (510–570 nm). Green light intensity increased due to the mycelium covering the crop surface (Graeff et al., 2006; Mahlein et al., 2013; Shen et al., 2015a) and a reduction in both chlorophyll content and light interception (Feng et al., 2016). In this study, correlation between disease severity with PWC and Chl content was significantly negative (*R*^2^ > 0.67). Although, 900–970 nm is the most sensitive band range of water absorption in near-infrared regions (Penuelas et al., 1993), the traditional water VIs as WBI, FWBI_1_, FWBI_2_ were unsuitable to detect the water status under powdery mildew stress. In this paper, relationships of the above three indices to water content possessed a weak correlation, and the precision was greatly reduced (*R*^2^ < 0.5). This may be due to a mixture phenomenon of physiological signal coverage and then confuse water absorption signal on canopy spectra under powdery mildew stress. Therefore, assessing PWC under powdery mildew stress using the traditional water indices (WBI, FWBI), is difficult at canopy level.

Plant disease influences tissue and organ physiological state and ultimately changes the reflective spectrum of plants (Jackson, 2003). Therefore, the disease sensitive waveband is reasonable for detecting plant physiological changes. Some researchers investigated the green waveband to form VIs for monitoring disease, such as dual-green vegetation index (Feng et al. 2016), powdery mildew index (Mahlein et al., 2013), and PRI (Huang et al. 2007). Spectral reflectance at 531 nm can provide an indicator of both photosynthetic function and interconversion of xanthophyll cycle pigments (Gamon et al., 1992). PRI was shown to track plant status in response to changing environmental conditions (Magney et al., 2016). In this paper, PRI (531,570) showed a good relationship with disease index (*R*^2^ = 0.649), and a significant relationship with PWC (*R*^2^ = 0.817) and fluorescence parameter (Fv/Fm, *R*^2^ = 0.833; Fv/Fo, *R*^2^ = 0.808). When white powdery mycelium covers the leaf surface, it causes a loss of pigment, fading leaves, and deteriorated leaf structure. These factors are closely linked with the green waveband. Reduced photosynthesis is closely linked to the green waveband in the visible range at canopy level (Evain et al., 2004). Gamon et al. (1992) proposed that PRI was an indicator of the de-epoxidation state of the xanthophyll cycle pigments and the efficiency of photosynthesis. Many studies have evaluated crop water stress based on xanthophyll compositional change (Penuelas et al., 1997a; Suárez et al., 2008, 2010; Neues et al., 2009; Zarco-Tejada et al., 2012), and the proportion of violaxanthin converted into zeaxanthin under stress conditions (Gamon et al., 1992). Previous research quantified relationships between PRI and physiological indicators, such as canopy temperature (Suárez et al., 2009), leaf stomatal conductance (Suárez et al., 2008), xylem water potential (Stagakis et al., 2012), relative leaf water content and the difference between leaf and air temperature (Rossini et al., 2013) and chlorophyll fluorescence parameters (Evain et al., 2004; Dobrowski et al., 2005). Therefore, PRI was proposed as an indicator of water stress (Suárez et al., 2008; Neues et al., 2009; Stagakis et al., 2012). When wheat is infected with powdery mildew, PWC and Chl contents decreased (Figure 6), and PRI increased obviously (Figure 7A). PRI showed a close correlation with PSII light use efficiency (F_v_/F_o_, F_v_/F_m_) under powdery mildew stress (Figures 7C,D). Unlike conventional water indices based on water absorption signal, PRI accurately tracked water content in infected wheat based on the relationships between PRI and growth parameters, and the PSII photochemical properties related to xanthophyll changed under biotic stress.

In remote sensing monitoring, factors affecting reflectivity should be considered, for example canopy structure, background, sun angle, leaf area index (LAI), and leaf angle distribution (LAD), etc. Previous studies have shown that the main factors affecting spectrum are LAI and LAD at the canopy level, and major changes are in the near-infrared region 760–900 nm (Dian and Fang, 2013). Under the same LAI, the reflectivity of the canopy is reduced with increasing average leaf angle (Zhao et al., 2009); the bigger the leaf angle, the more compact the plant, the weaker the ability to intercept light. There is a close relationship between PWC and plant morphological structure factors, changes in leaf angle ensure the plant can maximize moisture and light in the environment (Wu et al., 2014). Leaf water content of infected plants is usually reduced; conversely, leaf angle is increased, which may influence the reflectance spectrum in the near infrared bands (Cheng et al., 2010). When plants were infected, plant water metabolism was disturbed and PWC gradually decreased in our experiments. Therefore, the spectral reflectance of infected wheat was affected by comprehensive factors, such as water content, leaf angle and disease spot. In our studies, PRI successfully indicated PWC under two experimental systems with powdery mildew stress, and a corresponding model could reliably estimate PWC across different growth conditions. These results show that PRI is a viable indicator for detecting wheat water content under powdery mildew stress using an *in-situ* spectrometer. Of course, the detecting model was derived from a susceptible variety in two experimental conditions. Therefore, further analysis would be necessary to understand the effectiveness of PRI model monitoring of dynamic changes in plant water of various plant species under different disease stress.

## Conclusions

The physiological analysis of plant disease is extremely important for understanding relationships between VIs and growth parameters under biotic stress. Ground based remote sensing data acquired with the FieldSpec HandHeld spectrometer (Analytical Spectral Devices Inc., USA) were used to detect the variation of PWC of winter wheat infected with powdery mildew. The common water band indices, WBI, FWBI_1_, and FWBI_2_ were inappropriate for estimating the PWC under powdery mildew stress (*R*^2^ < 0.5). Physiological indicators (Chl content, PWC) significantly responded to increasing disease severity levels. The ordinal regressions between the PRI and plant physiological parameters under powdery mildew stress applied in the field, showed that PRI was sensitive to physiological change, such as Chl content (*R*^2^ = 0.639), Fv/Fm (*R*^2^ = 0.833), and Fv/Fo (*R*^2^ = 0.808). The green band range (510–570 nm) was valuable for detection of physiological parameters under the powdery mildew stress, and the unified relationship between PRI and PWC generated higher precision (*R*^2^ = 0.817), and lower error (RE = 8.26%). This showed that the PRI model has better compatibility between different test conditions and sampling periods, and PRI could be used as a plant water indicator of infected wheat at canopy level. This result will provide a theoretical basis for monitoring plant moisture dynamic variation under biotic stress in winter wheat using remote sensing technology.

## Author contributions

WF and XL conceivedand designed the research; WF and SQ analyzed the data and wrote the manuscript; YH, YW, WL, and YZ provided data and data acquisition capacity; and LH assisted manuscript writing and editing.

### Conflict of interest statement

The authors declare that the research was conducted in the absence of any commercial or financial relationships that could be construed as a potential conflict of interest.

## References

[B1] Allen P. J. (1942). Changes in the metabolism of wheat leaves induced by infection with powdery mildew. Am. J. Bot. 29, 425–435. 10.2307/2437306

[B2] Baker N. R. Rosenqvist E. (2004). Applications of chlorophyll fluorescence can improve crop production strategies: an examination of future possibilities. J. Exp. Bot. 55, 1607–1621. 10.1093/jxb/erh196 15258166

[B3] Bravo C. Moshou D. West J. McCartney A. Ramon H. (2003). Early disease detection in wheat fields using spectral reflectance. Biosyst. Eng. 84, 137–145. 10.1016/S1537-5110(02)00269-6

[B4] Cai C. J. Zhan-Hong M. A. Wang H. G. Zhang Y. P. Huang W. J. (2007). Comparison research of hyperspectral properties between near-ground and high altitude of wheat stripe rust. Acta Phytopathol. Sin. 37, 77–82. 10.3321/j.issn:0412-0914.2007.01.012

[B5] Cao X. R. Luo Y. Zhou Y. L. Duan X. Y. Cheng D. F. (2013). Detection of powdery mildew in two winter wheat cultivars using canopy hyperspectral reflectance. Crop Prot. 45, 124–131. 10.1016/j.cropro.2012.12.002 PMC4376796 25815468

[B6] Castro A. I. D. Ehsani R. Ploetz R. C. Crane J. H. Buchanon S. (2015). Detection of laurel wilt disease in avocado using low altitude aerial imaging. PLoS ONE 10:e0124642. 10.1371/journal.pone.0124642 25927209 PMC4415916

[B7] Castro A. I. D. Jurado-Expósito M. Gómez-Casero M. T. López-Granados F. (2012). Applying neural networks to hyperspectral and multispectral field data for discrimination of cruciferous weeds in winter crops. Sci. World J. 8, 1–11. 10.1100/2012/630390 PMC3354564 22629171

[B8] Chen B. Li S. K. Wang K. R. Wang F. Y. Xiao C. H. Pan W. C. (2010). Study on hyperspectral estimation of pigment contents in leaves of cotton under disease stress. Spectrosc. Spect. Anal. 30, 421–425. 10.3964/j.issn.1000-0593(2010)02-0421-05 20384137

[B9] Chen B. Wang K. R. Li S. K. Jin X. L. Chen J. L. Zhang D. S. (2011). The effects of disease stress on spectra reflectance and chlorophyll fluorescence characteristics of cotton leaves. J. Agr. Eng. Ers. 27, 86–93. 10.3969/j.issn.1002-6819.2011.09.017

[B10] Cheng S. X. Shao Y. N. Di W. U. Yong H. E. (2011). Determination of rice leaf blast disease level based on visible-near-infrared spectroscopy. J. Zhejiang Univ. 37, 307–311. 10.1631/jzus.C1000097

[B11] Cheng T. Rivard B. Sánchez-Azofeifa G. A. Feng J. Calvo-Polanco M. (2010). Continuous wavelet analysis for the detection of green attack damage due to mountain pine beetle infestation. Remote Sens. Environ. 114, 899–910. 10.1016/j.rse.2009.12.005

[B12] Christen D. Schönmann S. Jermini M. Strasser R. J. Défago G. (2007). Characterization and early detection of grapevine (*Vitis vinifera*) stress responses to esca disease by *in situ*, chlorophyll fluorescence and comparison with drought stress. Environ. Exp. Bot. 60, 504–514. 10.1016/j.envexpbot.2007.02.003

[B13] Christou P. Twyman R. M. (2004). The potential of genetically enhanced plants to address food insecurity. Nutr. Res. Rev. 17, 23–42. 10.1079/NRR200373 19079913

[B14] Dammer K. H. Möller B. Rodemann B. Heppner D. (2011). Detection of head blight (*Fusarium* ssp.) in winter wheat by color and multispectral image analyses. Crop Prot. 30, 420–428. 10.1016/j.cropro.2010.12.015

[B15] Dian Y. Y. Fang S. H. (2013). Simulation analysis of vegetation TOA reflectance based on coupled leaf-canopy-atmosphere radiative transfer model. Remote Sens. Land Res. 25, 30–37. 10.6046/gtzyyg.2013.03.06

[B16] Dobrowski S. Z. Pushnik J. C. Zarco-Tejada P. J. Ustin S. L. (2005). Simple reflectance indices track heat and water stress-induced changes in steady-state chlorophyll fluorescence at the canopy scale. Remote Sens. Environ. 97, 403–414. 10.1016/j.rse.2005.05.006

[B17] Evain S. Flexas J. Moya I. (2004). A new instrument for passive remote sensing: 2. Measurement of leaf and canopy reflectance changes at 531 nm and their relationship with photosynthesis and chlorophyll fluorescence. Remote Sens. Environ. 91, 175–185. 10.1016/j.rse.2004.03.012

[B18] Feng W. Guo B. B. Wang Z. J. He L. Song X. Wang Y. H. (2014). Measuring leaf nitrogen concentration in winter wheat using double-peak spectral reflection remote sensing data. Field Crop. Res. 159, 43–52. 10.1016/j.fcr.2014.01.010

[B19] Feng W. Shen W. Y. He L. Duan J. Z. Guo B. B. Li Y. (2016). Improved remote sensing detection of wheat powdery mildew using dual-green vegetation indices. Precis. Agric. 17, 608–627. 10.1007/s11119-016-9440-2

[B20] Feng W. Wang X. Y. Song X. He L. Wang C. Y. Guo T. C. (2013). Hyperspectral estimation of canopy chlorophyll density in winter wheat under stress of powdery mildew. J. Agr. Eng. Ers. 29, 114–123. 10.3969/j.issn.1002-6819.2013.13.016

[B21] Gamon J. A. Penuelas J. Field C. B. (1992). A narrow-waveband spectral index that tracks diurnal changes in photosynthetic efficiency. Remote Sens. Environ. 41, 35–44. 10.1016/0034-4257(92)90059-S

[B22] Gamon J. A. Surfus J. S. (1999). Assessing leaf pigment content and activity with a reflectometer. New Phytol. 143, 105–117. 10.1046/j.1469-8137.1999.00424.x

[B23] Gitelson A. A. Kaufman Y. J. Stark R. Rundquist D. (2002). Novel algorithms for remote estimation of vegetation fraction. Remote Sens. Environ. 80, 76–87. 10.1016/S0034-4257(01)00289-9

[B24] Graeff S. Link J. Claupein W. (2006). Identification of powdery mildew (*Erysiphe graminis* sp. tritici) and take-all disease (*Gaeumannomyces graminis* sp. tritici) in wheat (*Triticum aestivum* L.) by means of leaf reflectance measurements. Cent. Eur. J. Biol. 1, 275–288. 10.2478/s11535-006-0020-8

[B25] Harris A. Bryant R. G. Baird A. J. (2006). Mapping the effects of water stress on Sphagnum: preliminary observations using airborne remote sensing. Remote Sens. Environ. 100, 363–378. 10.1016/j.rse.2005.10.024

[B26] Huang W. J. David W. L. Zheng N. Zhang Y. J. Liu L. Y. Wang J. H. (2007). Identification of yellow rust in wheat using in-situ spectral reflectance measurements and airborne hyperspectral imaging. Precis. Agric. 8, 187–197. 10.1007/s11119-007-9038-9

[B27] Jackson R. D. (2003). Remote sensing of biotic and abiotic plant stress. Annu. Rev. Phytopat. 24, 265–287. 10.1146/annurev.py.24.090186.001405

[B28] Janka E. Körner O. Rosenqvist E. Ottosen C. O. (2013). High temperature stress monitoring and detection using chlorophyll a fluorescence and infrared thermography in chrysanthemum (*dendranthema grandiflora*). Plant Physiol. Biochem. 67, 87–94. 10.1016/j.plaphy.2013.02.025 23545206

[B29] Jones C. L. Weckler P. R. Maness N. O. Stone M. L. Jayasekara R. (2004). Estimating water stress in plants using hyperspectral sensing. System 10.13031/2013.17087

[B30] Larsolle A. Muhammed H. H. Stafford J. V. (2007). Measuring crop status using multivariate analysis of hyperspectral field reflectance with application to disease severity and plant density. Precis. Agric. 8, 37–47. 10.1007/s11119-006-9027-4

[B31] Li R. H. Guo P. G. Baum M. Grando S. Ceccarelli S. (2006). Evaluation of chlorophyll content and fluorescence parameters as indicators of drought tolerance in barley. J. Integr. Agr. 5, 751–757. 10.1016/s1671-2927(06)60120-x

[B32] Lichtenthaler H. K. (1987). Chlorophylls and carotenoids: pigments of photosynthetic biomembranes. Method. Enzymol. 148, 350–382. 10.1016/0076-6879(87)48036-1

[B33] Lima J. D. Mosquim P. R. Da Matta F. M (1999). Leaf gas exchange and chlorophyll fluorescence parameters in *Phaseolus vulgaris* as affected by nitrogen and phosphorus deficiency. Photosynthetica 37, 113–121 10.1023/A:1007079215683

[B34] Liu L. Y. Huang W. J. Rui-Liang P. U. Wang J. H. (2014). Detection of internal leaf structure deterioration using a new spectral ratio index in the near-infrared shoulder region. J. Integr. Agr. 13, 760–769. 10.1016/S2095-3119(13)60385-8

[B35] Liu L. Y. Song X. Y. Li C. J. Qi L. Huang W. J. Wang J. H. (2009). Monitoring and evaluation of the diseases of and yield winter wheat from multi-temporal remotely-sensed data. J. Agr. Eng. Ers. 25, 137–143.

[B36] Magney T. S. Vierling L. A. Eitel J. U. H. Huggins D. R. Garrity S. R. (2016). Response of high frequency Photochemical Reflectance Index (PRI) measurements to environmental conditions in wheat. Remote Sens. Environ. 173, 84–97. 10.1016/j.rse.2015.11.013

[B37] Mahlein A. K. Rumpf T. Welke P. Dehne H. W. Plümer L. Steiner U. (2013). Development of spectral indices for detecting and identifying plant diseases. Remote Sens. Environ. 128, 21–30. 10.1016/j.rse.2012.09.019

[B38] Neues A. D. A. Poitevin C. A. Sloten J. V. D. Meerbeek B. V. Oosterwyck H. V. (2009). Modelling PRI for water stress detection using radiative transfer models. Remote Sens. Environ. 113, 730–744. 10.1016/j.rse.2008.12.001

[B39] Peixoto P. H. P. Da Matta F. M. Cambraia J. (2002). Responses of the photosynthetic apparatus to aluminium stress in two sorghum cultivars. J. Plant Nutr. 25, 821–832. 10.1081/PLN-120002962

[B40] Penuelas J. Filella I. Biel C. Serrano L. Save R. (1993). The reflectance at the 950–970 nm region as an indicator of plant water status. Int. J. Remote Sens. 14, 1887–1905. 10.1080/01431169308954010

[B41] Penuelas J. Gamon J. A. Fredeen A. L. Merino J. Field C. B. (1994). Reflectance indices associated with physiological changes in nitrogen- and water-limited sunflower leaves. Remote Sens. Environ. 48, 135–146. 10.1016/0034-4257(94)90136-8

[B42] Penuelas J. Llusia J. Pinol J. Filella I. (1997a). Photochemical reflectance index and leaf photosynthetic radiation-use-efficiency assessment in Mediterranean trees. Int. J. Remote Sens. 18, 2863–2868.

[B43] Penuelas J. Pinol J. Ogaya R. Filella I. (1997b). Estimation of plant water concentration by the reflectance Water Index WI (R900/R970). Int. J. Remote Sens. 18, 2869–2875.

[B44] Rossini M. Fava F. Cogliati S. Meroni M. Marchesi A. Panigada C. (2013). Assessing canopy PRI from airborne imagery to map water stress in maize. ISPRS J. Photogramm. Remote Sens. 86, 168–177. 10.1016/j.isprsjprs.2013.10.002

[B45] Sankaran S. Mishra A. Ehsani R. Davis C. (2010). A review of advanced techniques for detecting plant diseases. Comput. Electron. Agr. 72, 1–13. 10.1016/j.compag.2010.02.007

[B46] Sharma D. K. Fernández J. O. Rosenqvist E. Ottosen C. Andersen S. B. (2014). Genotypic response of detached leaves versus intact plants for chlorophyll fluorescence parameters under high temperature stress in wheat. J. Plant Physiol. 171, 576–586. 10.1016/j.jplph.2013.09.025 24709148

[B47] Shen W. Y. Feng W. Li X. Wang X. Y. He L. Guo T. C. Li Y. X. (2015a). Estimation model of wheat powdery mildew severity based on leaves hyperspectral characteristics. J. Triticeae Crops 35, 129–137. 10.7606/j.issn.1009-1041.2015.01.20

[B48] Shen W. Y. Li Y. Feng W. Zhang H. Y. Zhang Y. Xie Y. X. (2015b). Inversion model for severity of powdery mildew in wheat leaves based on factor analysis-BP neural network. J. Agr. Eng. Ers. 31, 183–190. 10.11975/j.issn.1002-6819.2015.22.025

[B49] Shirke P. A. Pathre U. V. (2003). Diurnal and seasonal changes in photosynthesis and photosystem 2 photochemical efficiency in prosopis juliflora, leaves subjected to natural environmental stress. Photosynthetica 41, 83–89. 10.1023/A:1025864513663

[B50] Sindhuja S. Ashish M. Reza E. Cristina D. (2010). A review of advanced techniques for detecting plant diseases. Comput. Electron. Agr. 72, 1–13. 10.1016/j.compag.2010.02.007

[B51] Stagakis S. González-Dugo V. Cid P. Guillén-Climent M. L. Zarco-Tejada P. J. (2012). Monitoring water stress and fruit quality in an orange orchard under regulated deficit irrigation using narrow-band structural and physiological remote sensing indices. ISPRS J. Photogramm. 71, 47–61. 10.1016/j.isprsjprs.2012.05.003

[B52] Steddom K. Heidel G. Jones D. Rush C. M. (2003). Remote detection of rhizomania in sugar beets. Phytopathology 93, 720–726. 10.1094/PHYTO.2003.93.6.720 18943059

[B53] Strachan I. B. Pattey E. Boisvert J. B. (2002). Impact of nitrogen and environmental conditions on corn as detected by hyperspectral reflectance. Remote Sens. Environ. 80, 213–224. 10.1016/S0034-4257(01)00299-1

[B54] Strange R. N. Scott P. R. (2005). Plant disease: a threat to global food security. Phytopathology 43, 83–116. 10.1146/annurev.phyto.43.113004.133839 16078878

[B55] Suárez L. Zarcotejada P. J. Berni J. A. J. Gonzálezdugo V. Fereres E. (2009). Modelling PRI for water stress detection using radiative transfer models. Remote Sens. Environ. 113, 730–744. 10.1016/j.rse.2008.12.001

[B56] Suárez L. Zarcotejada P. J. Gonzálezdugo V. Berni J. A. J. Sagardoy R. Morales F. (2010). Detecting water stress effects on fruit quality in orchards with time-series PRI airborne imagery. Remote Sens. Environ. 114, 286–298. 10.1016/j.rse.2009.09.006

[B57] Suárez L. Zarco-Tejada P. J. Sepulcre-Cantó G. Pérez-Priego O. Miller J. R. Jiménez-Mu-oz J. C. (2008). Assessing canopy PRI for water stress detection with diurnal airborne imagery. Remote Sens. Environ. 112, 560–575. 10.1016/j.rse.2007.05.009

[B58] Thenot F. Méthy M. Winkel T. (2002). The photochemical reflectance index (PRI) as a water-stress index. Int. J. Remote Sens. 23, 5135–5139 10.1080/01431160210163100

[B59] Tian Y. C. Yao X. Yang J. Cao W. X. Hannaway D. B. Zhu Y. (2011). Assessing newly developed and published vegetation indices for estimating rice leaf nitrogen concentration with ground- and space-based hyperspectral reflectance. Field Crops Res. 120, 299–310. 10.1016/j.fcr.2010.11.002

[B60] Tilley D. R. Ahmed M. Ji H. S. Badrinarayanan H. (2003). Hyperspectral reflectance of emergent macrophytes as an indicator of water column ammonia in an oligohaline, subtropical marsh. Ecol. Eng. 21, 153–163. 10.1016/j.ecoleng.2003.10.004

[B61] Usha K. Singh B. (2013). Potential applications of remote sensing in horticulture—a review. Sci. Hortic. 153, 71–83. 10.1016/j.scienta.2013.01.008

[B62] Wang D. Dowell F. E. Lan Y. Pasikatan M. Maghirang E. (2002). Determining pecky rice kernels using visible and near-infrared spectroscopy. Int. J. Food Prop. 5, 629–639. 10.1081/JFP-120015497

[B63] Wu N. Zhang Y. M. Downing A. Aanderud Z. T. Tao Y. Williams S. (2014). Rapid adjustment of leaf angle explains how the desert moss, *Syntrichia caninervis*, copes with multiple resource limitations during rehydration. Funct. Plant Biol. 41, 168–177. 10.1071/FP13054 32480976

[B64] Xu H. C. Luo Y. Q. Zhang T. T. Shi Y. J. (2011). Changes of reflectance spectra of pine needles in different stage after being infected by pine wood nematode. Spec. Spect. Anal. 31, 1352–1356. 10.3964/j.issn.1000-0593(2011)05-1352-05 21800599

[B65] Xu H. R. Ying Y. B. Fu X. P. Zhu S. P. (2007). Near-infrared spectroscopy in detecting leaf miner damage on tomato leaf. Biosyst. Eng. 96, 447–454. 10.1016/j.biosystemseng.2007.01.008

[B66] Zarco-Tejada P. J. González-Dugo V. Berni J. A. J. (2012). Fluorescence, temperature and narrow-band indices acquired from a UAV platform for water stress detection using a micro-hyperspectral imager and a thermal camera. Remote Sens. Environ. 117, 322–337. 10.1016/j.rse.2011.10.007

[B67] Zhang D. Y. Zhang J. C. Zhu D. Z. Wang J. H. Luo J. H. Zhao J. L.. (2011). Investigation of the hyperspectral image characteristics of wheat leaves under different stress. Spectrosc. Spect. Anal. 31, 1101–1105. 10.3964/j.issn.1000-0593(2011)04-1101-05 21714269

[B68] Zhang J. C. Pu R. L. Huang W. J. Yuan L. Luo J. H. Wang J. H. (2012a). Using *in-situ* hyperspectral data for detecting and discriminating yellow rust disease from nutrient stresses. Field Crop. Res. 134, 165–174. 10.1016/j.fcr.2012.05.011

[B69] Zhang J. C. Pu R. L. Wang J. H. Huang W. J. (2012b). Detecting powdery mildew of winter wheat using leaf level hyperspectral measurements. Comput. Electron. Agr. 85, 13–23. 10.1016/j.compag.2012.03.006

[B70] Zhang J. C. Pu R. L. Yuan L. Wang J. H. Huang W. J. Yang G. J. (2014). Monitoring powdery mildew of winter wheat by using moderate resolution multi-temporal satellite imagery. PLoS ONE 9:e93107. 10.1371/journal.pone.0093107 24691435 PMC3972229

[B71] Zhang M. Qin Z. Liu X. (2005). Remote sensed spectral imagery to detect late blight in field tomatoes. Precis. Agric. 6, 489–508. 10.1007/s11119-005-5640-x

[B72] Zhao C. J. Huang W. J. Mu X. H. Wang J. D. Wang J. H. (2009). Crop geometry identification based on inversion of semiempirical BRDF models. Spectrosc. Spect. Anal. 9, 2555–2559. 10.3964/j.issn.1000-0593(2009)09-2555-05 19950674

